# A Phosphorylatable Sphingosine Analog Induces Airway Smooth Muscle Cytostasis and Reverses Airway Hyperresponsiveness in Experimental Asthma

**DOI:** 10.3389/fphar.2017.00078

**Published:** 2017-02-21

**Authors:** David R. Gendron, Pascale B. Lecours, Anne-Marie Lemay, Marie-Josée Beaulieu, Carole-Ann Huppé, Audrey Lee-Gosselin, Nicolas Flamand, Anthony S. Don, Élyse Bissonnette, Marie-Renée Blanchet, Mathieu Laplante, Sylvain G. Bourgoin, Ynuk Bossé, David Marsolais

**Affiliations:** ^1^Centre de Recherche de l’Institut Universitaire de Cardiologie et de Pneumologie de Québec, QuébecQC, Canada; ^2^Faculty of Medicine, Université Laval, QuébecQC, Canada; ^3^Centenary Institute and NHMRC Clinical Trials Centre, University of Sydney, CamperdownNSW, Australia; ^4^Division of Infectious Diseases and Immunology, CHU de Québec Research Center, QuébecQC, Canada

**Keywords:** airway smooth muscle, sphingosine-1-phosphate, asthma, proliferation, airway hyperresponsiveness, sphingosine analog

## Abstract

In asthma, excessive bronchial narrowing associated with thickening of the airway smooth muscle (ASM) causes respiratory distress. Numerous pharmacological agents prevent experimental airway hyperresponsiveness (AHR) when delivered prophylactically. However, most fail to resolve this feature after disease is instated. Although sphingosine analogs are primarily perceived as immune modulators with the ability to prevent experimental asthma, they also influence processes associated with tissue atrophy, supporting the hypothesis that they could interfere with mechanisms sustaining pre-established AHR. We thus assessed the ability of a sphingosine analog (AAL-R) to reverse AHR in a chronic model of asthma. We dissected the pharmacological mechanism of this class of agents using the non-phosphorylatable chiral isomer AAL-S and the pre-phosphorylated form of AAL-R (AFD-R) *in vivo* and in human ASM cells. We found that a therapeutic course of AAL-R reversed experimental AHR in the methacholine challenge test, which was not replicated by dexamethasone or the non-phosphorylatable isomer AAL-S. AAL-R efficiently interfered with ASM cell proliferation *in vitro*, supporting the concept that immunomodulation is not necessary to interfere with cellular mechanisms sustaining AHR. Moreover, the sphingosine-1-phosphate lyase inhibitor SM4 and the sphingosine-1-phosphate receptor antagonist VPC23019 failed to inhibit proliferation, indicating that intracellular accumulation of sphingosine-1-phosphate or interference with cell surface S1P_1_/S1P_3_ activation, are not sufficient to induce cytostasis. Potent AAL-R-induced cytostasis specifically related to its ability to induce intracellular AFD-R accumulation. Thus, a sphingosine analog that possesses the ability to be phosphorylated *in situ* interferes with cellular mechanisms that beget AHR.

## Introduction

Asthma is a prevalent and complex syndrome (Lotvall et al., 2011). Alarmingly, a significant proportion of asthmatic patients responds poorly to mainstay therapies, some achieving symptom control only with oral prednisone; and others remaining refractory to any pharmacological option currently available (Reddel et al., 2015). There is thus a dire need for new therapeutic options in asthma.

One of the distinctive features of asthma is AHR. Among the multiple factors that interact to elicit AHR, the increased amount of smooth muscle within the airway wall is thought to contribute significantly. This paradigm is fuelled by several observations. First, the amount of ASM is positively correlated with the severity of asthma (James et al., 2009). Second, the capacity to generate stress (force per cross-sectional area) is identical between asthmatic and non-asthmatic ASM tissues (Ijpma et al., 2015), implying that the amount rather that the stress-generating capacity of ASM is involved in AHR. Third, airway narrowing measured directly *ex vivo* is determined by the amount of ASM (Noble et al., 2013). Strategies that aim at reducing the amount of ASM should benefit asthmatic patients by reducing their level of airway responsiveness. In this regard, bronchial thermoplasty, an interventional procedure proven to reduce ASM volume (Salem et al., 2016), decreases by nearly 50% the rate of exacerbations in patients with moderate to severe persistent asthma (Wechsler et al., 2013). However, there is currently no pharmacological treatment that efficiently reverses ASM thickening.

**FIGURE 1:**
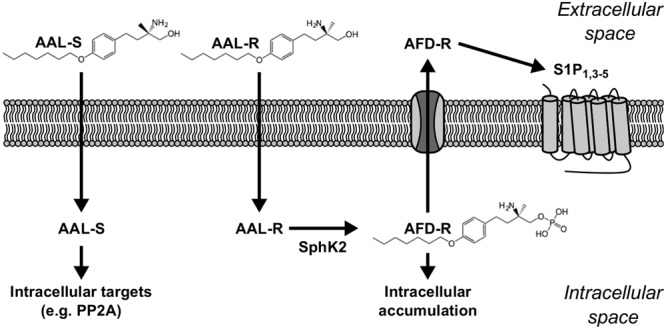
**Sphingosine analogs and their targets.** AAL-R [(*R*)-2-amino-4-(4-heptyloxyphenyl)-2-methylbutanol] and AAL-S [(*S*)-2-amino-4-(4-heptyloxyphenyl)-2-methylbutanol] are cell-permeant sphingosine analogs that readily penetrate into the intracellular space. Inside the cell, AAL-R is rapidly phosphorylated by SphK2 into AFD-R [phosphorylated (*R*)-2-amino-4-(4-heptyloxyphenyl)-2-methylbutanol]. Although not phosphorylatable, AAL-S still has biological relevancy, such as positive regulatory effects on intracellular Protein Phosphatase 2A (PP2A) (Collison et al., 2013). The cell-impermeant molecule AFD-R can either accumulate intracellularly to exert biological effects via unknown targets (Jary et al., 2010), or be transported into the extracellular space where it can modulate cell surface S1P receptors.

Classically, sphingosine analogs like FTY720 and AAL-R were recognized as S1P receptor modulators on the basis of their propensity to be phosphorylated *in vivo* by SphK2, and to induce S1P receptor-mediated events including lymphopenia and bradycardia (Mandala et al., 2002; Sanna et al., 2004). Yet, an emerging body of evidence now supports the concept that sphingosine analogs may counteract mechanisms that sustain or promote ASM thickening. For instance the non-phosphorylatable sphingosine analog AAL-S reverses the inhibition of PP2A (**Figure 1**) (Collison et al., 2013; Rahman et al., 2016b) that occurs in allergic airways, which could potentially interfere with proliferative cascades associated with ASM cell expansion. Other evidence suggests that high doses of the sphingosine analog FTY720 influence the levels of ceramides (Oyeniran et al., 2015), which are potent inducers of apoptosis and modulators of chronic inflammation. In addition, intracellular S1P can cause autophagy (Liao et al., 2012) and inhibit proliferation (Liao et al., 2007), which in turn, could counteract ASM thickening (Stamatiou et al., 2009). Importantly, *in vitro* data show that sphingosine analogs can counteract cell amplification and/or survival via SphK2-dependent intracellular accumulation of phosphorylated sphingoid bases (Don et al., 2007; Jary et al., 2010). In agreement with this growing body of evidence, we tested the concept that this class of agents could reverse ASM thickening. We show that the sphingosine analog AAL-R efficiently reverses AHR and the underlying thickening of ASM. We unravel the potential for this class of agents to act directly on ASM cells and to induce cytostasis by a mechanism featuring intracellular conversion of AAL-R to its phosphorylated form.

## Materials and Methods

### Murine Model and Experimental Treatments

**FIGURE 2:**
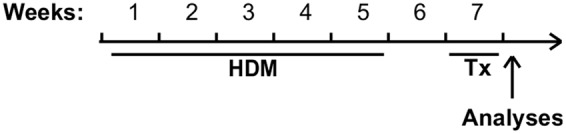
**Experimental model.** Mice received either saline or HDM three times a week for 5 consecutive weeks. After 1 week of rest to allow acute inflammation to resolve, mice were administered pharmacological treatments daily for a period of 1 week and euthanized 24 h after the last dose for analyses.

Pathogen-free BALB/c female mice (8 weeks old, Charles River, Saint-Constant, QC, Canada) randomly assigned to experimental groups were anesthetized with isoflurane and instilled intranasally with 50 μl of saline or with saline containing 50 μg of HDM extract (*D. pteronyssinus*; Greer, Lenoir, NC, USA), three times a week, for 5 weeks. Mice were then left untouched during week 6 (**Figure 2**). Sphingosine analogs (AAL-S or AAL-R; see Gendron et al., 2015) or dexamethasone (Sigma Aldrich, Oakville, ON, Canada) were injected i.p. at a dose of 1 mg/kg once daily from day 7 to day 14 following the last HDM instillation, and compared with a group receiving an equal volume of the vehicle. Twenty-four hours following the last injection, mice were anesthetized with ketamine-xylazine and loss of toe-pinch reflex was verified. Mice were then tracheotomised and connected to the flexiVent apparatus (SCIREQ, Montreal, QC, Canada) to measure the level of airway responsiveness to methacholine (OMEGA, Montreal, QC, Canada) as described previously (Blanchet et al., 2007). To avoid artifacts due to spontaneous respiratory muscle contractions, mice were paralyzed with pancuronium (Sandoz, Boucherville, QC, Canada). Heart rate was monitored until euthanasia in order to ensure proper anesthesia. Bronchoalveolar lavage fluid was harvested as described (Gendron et al., 2015) and tissues were then fixed in phosphate buffered saline containing 4% paraformaldehyde for histological analyses. Experiments, housing and care procedures were approved by the Committee of Animal Care of Université Laval in accordance with the guidelines of the Canadian Council on Animal Care (protocol 2013037).

### Histological Analyses

Five micrometers-thick paraffin-embedded transverse lung slices were stained with Masson’s trichrome for quantification of ASM thickness. Using NDP View (Hamamatsu Photonics, Hamamatsu City, Japan), the length of the basal membrane and the area of ASM for individual circular bronchi was measured. ASM thickness was defined as area of ASM divided by the length of the basal membrane. Two to five bronchi were measured blindly per lung and the average size of bronchi did not differ between groups.

### Cell Culture

Human primary ASM cells (CC-2576; Lonza, Walkersville, MD, USA) were plated at a density of 10^4^ cells per cm^2^ in Dulbecco’s Modified Eagle Medium containing 10% fetal bovine serum (complete medium) for 24 h, and then incubated with sphingosine analogs, the S1P lyase inhibitor (SM4; Zhao et al., 2015) or the dual S1P receptor (S1P_1-3_) antagonist (VPC23019; Tocris, Burlington, ON, Canada) for 24–72 h. Cells were either incubated with tetrazolium dye (MTT assay; 3-(4,5-dimethylthiazol-2-yl)-2,5-diphenyltetrazolium bromide; Sigma Aldrich, Oakville, ON, Canada) to determine the viable cell numbers; or incubated with bromodeoxyuridine (BrdU; 5-Bromo-2′-deoxyuridine; Roche, Laval, QC, Canada) overnight to assess proliferation. Flow cytometric analyses were performed as described (Gendron et al., 2015).

### Quantification of Sphingolipids

Cell pellets were spiked with internal standards (C17-sphingosine Cayman Chemical, Ann Arbor, MI, USA; C17-S1P, Avanti Polar Lipids, Alabaster, AL, USA) and lipids were extracted using a published method (Couttas et al., 2014). Liquid chromatography tandem-mass spectrometry was performed at PhenoSwitch Bioscience, Inc. (Sherbrooke, QC, Canada). Liquid chromatography gradient was performed using 0.2% v/v formic acid in water and with 0.2% v/v formic acid with 10 mM ammonium formate in methanol on a reversed phase Halo PFP column (Advance Materials Technology, Wilmington, DE, USA), which was maintained at 50°C. Acquisition was performed with an ABSciex TripleTOF 5600 (Sciex, Foster City, CA, USA) equipped with an electrospray interface. Quantification was done using the area under the curve with the MuliQuant software (Sciex). See Supplementary Methods and Supplementary Table 1 for details.

### Statistics

Data were expressed using mean ± SEM. Homogeneity of variance and normality of the data were verified. When appropriate, variables were log-transformed. Rank transformation and the ordinary F test from ANOVA were used when appropriate. Data from mice were analyzed using ANOVAs or two-way ANOVAs with an interaction effect. When heteroscedasticity was significant, the statistical analyses were performed using separate residual variance per group. The Satterthwaite’s degree of freedom statement was added for unequal variance structure. Posteriori comparisons were performed using Tukey’s comparison. When appropriate, bi-directional unpaired *t*-test was employed. The results were considered significant with *p*-values ≤ 0.05. Data were analyzed in collaboration with a professional biostatistician at the IUCPQ biostatistics core service using the statistical package program SAS v9.4 (RRID:SCR_008567; SAS Institute, Inc., Cary, NC, USA).

## Results

### AAL-R Reverses Airway Hyperresponsiveness and Airway Smooth Muscle Thickening

**FIGURE 3:**
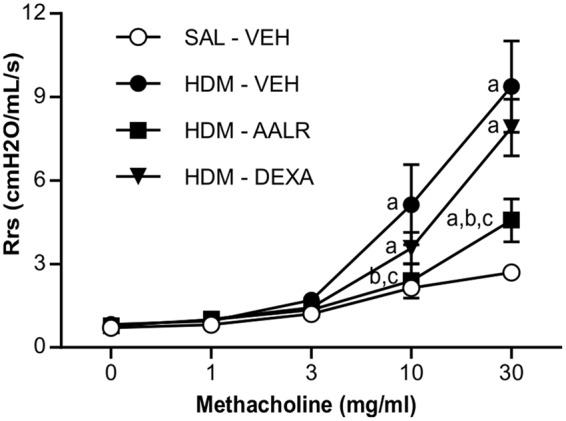
**AAL-R reverses airway hyperresponsiveness elicited by chronic HDM exposure.** Saline- or HDM- treated mice were administered vehicle (VEH), AAL-R (1 mg/kg) or Dexamethasone (DEXA) (1 mg/kg) and the degree of airway responsiveness was assessed by measuring the resistance of the airway system (Rrs) in response to graded doses of methacholine. *n* = 8 mice per group except DEXA where *n* = 6. “a” denotes a significant difference vs. the SAL – VEH group, “b”: vs. HDM – VEH group, “c”: vs. HDM – DEXA group. Significantly different at *p* < 0.05.

Using a model where persistent ASM thickening and AHR are pre-established using chronic exposure to HDM (**Figure 2**), we first evaluated the *in vivo* potential of AAL-R to reverse AHR, when administered at well-tolerated doses, yet sufficient to induce biological effects. We found that AAL-R (1 mg/kg/day) reversed HDM-induced AHR to a level 70% lower than the vehicle treatment (**Figure 3**). Contrary to AAL-R, corticosteroid intervention (dexamethasone; 1 mg/kg/day) failed to reverse AHR, furthering the concept of usefulness for sphingosine analogs in the context of asthma. On that note, we previously showed that AAL-R efficiently interfered with the inflammatory cascade in response to HDM soon after its instatement in the airways (Gendron et al., 2015). In face of differing immune mechanisms occurring in the remodeled airways (Mushaben et al., 2013; Sordillo and Raby, 2014), and since alleviation of eosinophilic inflammation can interfere with pathogenic mechanisms of asthma, we also confirmed that AAL-R blunted the antigen rechallenge-induced lymphocyte accumulation in the bronchoalveolar lavage fluid, which was accompanied by a profound 54% decrease of eosinophil numbers (Supplementary Figure 1).

**FIGURE 4:**
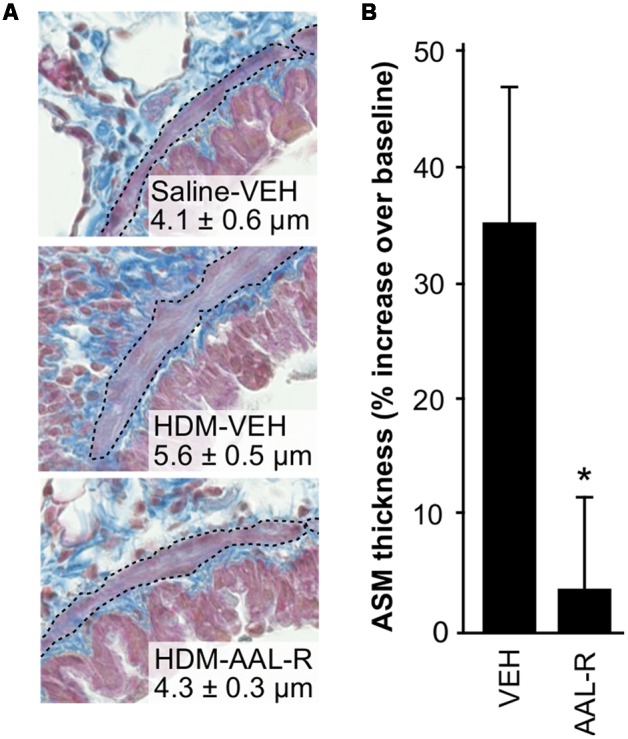
**AAL-R reverses ASM thickening.** Mice received HDM or saline (SAL) and underwent VEH or AAL-R treatment as described in **Figure 2**. **(A)** Histological appearance of the airway wall. The smooth muscle layer is delineated with a black dotted line. One representative image per group is shown and the numbers represent the average thickness of the ASM layer ± SEM. **(B)** The percentage of increase in ASM thickness relatively to saline-VEH mice (baseline) was computed. Saline: *n* = 4; VEH: *n* = 7; AAL-R: *n* = 7; ^∗^Significantly different at *p* < 0.05.

Since ASM thickening is central to the development of AHR, we performed a new series of experiments where lungs of mice receiving either AAL-R or vehicle after chronic HDM exposure were stained with Masson’s trichrome, in order to visualize the ASM layer located between the epithelium and the parenchyma (**Figure 4A**). Mice receiving saline and vehicle were used as baseline controls. Planimetry on transverse lung slices revealed an average bronchial smooth muscle thickness of 4.1 ± 0.6 μm in the group receiving saline and vehicle. In the group receiving HDM and vehicle, ASM thickness was increased to 5.6 ± 0.5 μm. This 36% increase in ASM thickness (**Figure 4B**) was reduced to a near-baseline level of 4.3 ± 0.3 μm by AAL-R. In agreement with reduced ASM thickness, AAL-R again robustly reversed AHR caused by chronic exposure to HDM (not shown). Thus, these three series of experiments revealed that AAL-R retains its ability to inhibit TH2 inflammation in the remodeled airways and efficiently reverses ASM thickening as well as the ensuing AHR.

### AAL-R Favors an Anti-proliferative Balance in ASM Cells

**FIGURE 5:**
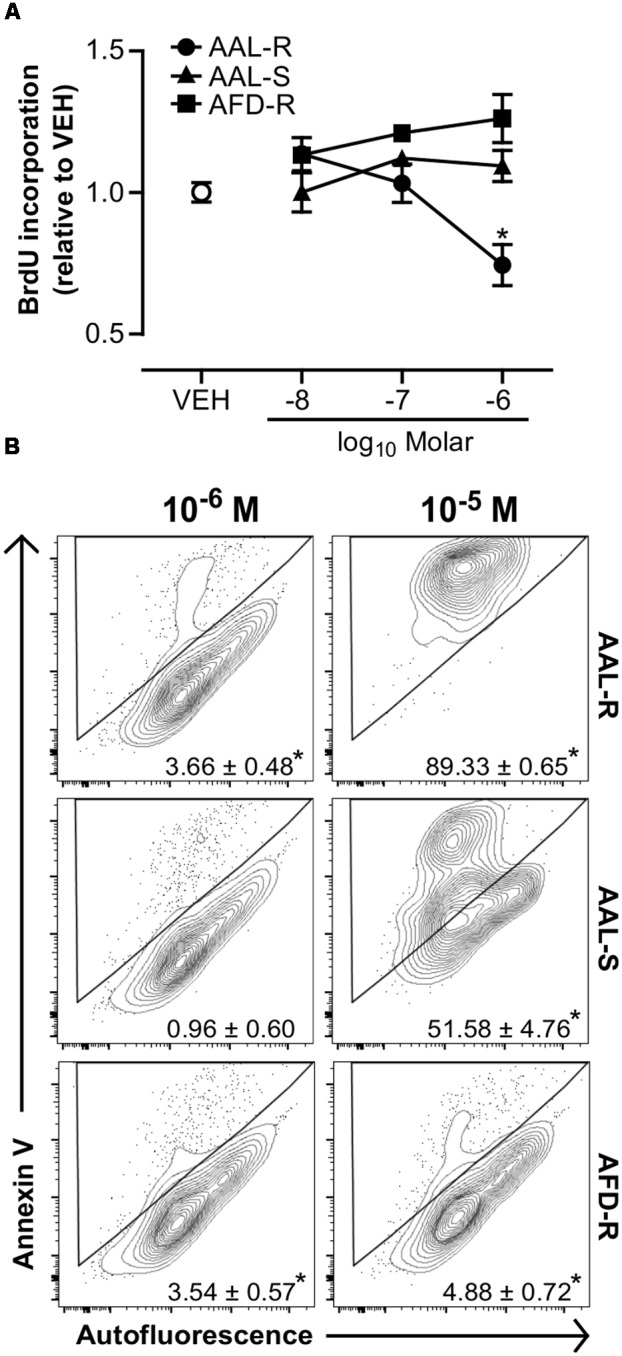
**AAL-R inhibits the proliferation of ASM cells.** ASM cells were incubated in complete medium with the indicated concentrations of AAL-R, AAL-S, AFD-R or the vehicle (VEH; empty dot) for 72 h. **(A)** Proliferation was assessed by bromodeoxyuridine incorporation. *n* = 4; ^∗^significantly different at *p* < 0.05. **(B)** Annexin V binding was evaluated after 48 h of incubation. Numbers show drug-induced increase in apoptosis (percentage over vehicle-treated baseline). *n* = 6 except for VEH: *n* = 9 and AAL-R 10^-6^ M: *n* = 8. ^∗^Shows statistical significance from VEH group at a *p* < 0.05. All panels show representative results from two independent experiments.

Because AAL-R reverses AHR when administered at a phase where acute inflammation is resolved, we next addressed the idea that AAL-R could act directly on ASM cells to impair underlying features of ASM thickening. We show that AAL-R, but not its pre-phosphorylated/cell impermeant form (AFD-R), nor its non-phosphorylatable isomer (AAL-S), efficiently interfered with the proliferation of ASM. For instance, AAL-R induced a 26% inhibition of bromodeoxyuridine incorporation at 1 μM (**Figure 5A**), when compared to the vehicle. We then set out to determine whether apoptosis also contributed to the inhibition of ASM accumulation under mitogenic conditions. We found that, at concentrations of 1 μM or lower, AAL-R mildly modulated apoptosis *in vitro* (**Figure 5B**), while being a potent cell death inducer at 10 μM with nearly 100% dead cells. On the other hand, AFD-R failed to induce apoptosis, even at the concentration of 10 μM (**Figure 5B**), and AAL-S only induced partial apoptosis at 10 μM. Our results thus unravel a clear potential for AAL-R to interfere with mechanisms that sustain ASM cell proliferation.

### Modulation of S1P Levels Does Not Resolve the Potent Anti-proliferative Effect of AAL-R

**FIGURE 6:**
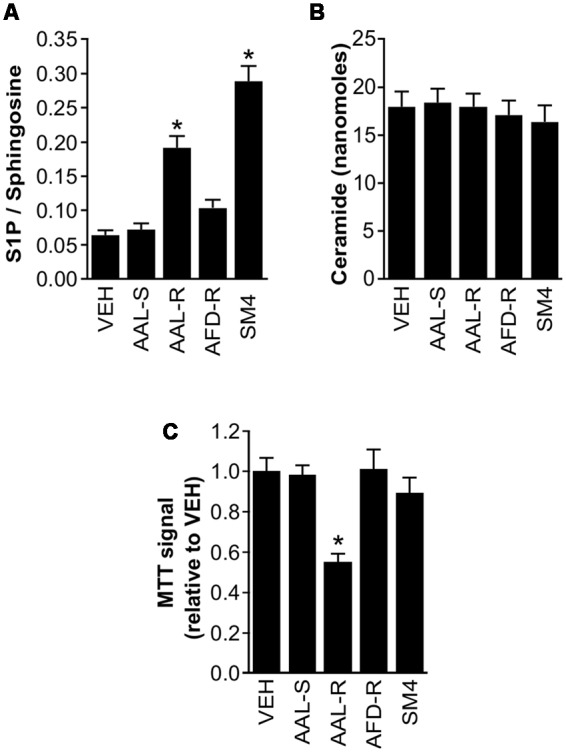
**Endogenous sphingolipid modulation in response to sphingolipid modifiers.** ASM cells were incubated in complete medium. When indicated, AAL-R (1 μM), AAL-S (1 μM), AFD-R (1 μM), SM4 (3 μM) or the vehicle (VEH) were added for 24 h. Mass spectrometry was performed to quantify S1P, sphingosine and ceramide in ASM cells. Shown is **(A)** the S1P/sphingosine ratio and **(B)** C-16 ceramide. **(C)** Modulation of cell accumulation in response to the experimental agents measured using the tetrazolium dye assay. *n* = 6. ^∗^Shows statistically significant differences from the VEH group at a *p* < 0.05.

Given the putative impact of sphingosine analogs on the balance of sphingolipidic species, we tested the concept that intracellular S1P levels could explain the differential potency of sphingosine analogs in inducing ASM cytostasis. We first characterized which S1P pathway proteins were expressed in ASM cells (Supplementary Figure 2). We detected the expression of *S1PR1* to *S1PR3*, both sphingosine kinases and S1P lyase. In agreement with previously-documented sphingosine and S1P levels in biological tissues, we found approximately 10 times more sphingosine than S1P in our ASM cultures (**Figure 6**). AAL-R increased absolute levels of intracellular S1P (2.0 ± 0.13 picomoles) when compared to the vehicle (0.5 ± 0.05 picomoles), to AAL-S (0.6 ± 0.06 picomoles), and to AFD-R (0.9 ± 0.05 picomoles). This resulted in a significant twofold increase in the S1P/sphingosine balance, when compared to the vehicle treatment (**Figure 6A**).

To resolve whether or not increase of intracellular S1P was sufficient to induce cytostatis, cells were incubated with an inhibitor of S1P lyase (Zhao et al., 2015) (**Figure 6**). The S1P lyase inhibitor SM4 potently increased intracellular S1P level (2.8 ± 0.08 picomoles), which resulted in a 33% increase of the S1P/sphingosine ratio compared to the AAL-R-treated cells (**Figure 6A**). This occurred in the absence of an effect on the C16-ceramide content (**Figure 6B**). Also, we found no evidence that AAL-R, AAL-S, or AFD-R (at the concentration of 1 μM) impacted on cellular levels of C16-ceramide, arguing against a major role for a shift in the sphingolipid rheostat to justify AAL-R-induced cytostasis. In spite of potent enhancement of intracellular S1P content, SM4 failed to inhibit ASM cell proliferation (**Figures 6A,C**), revoking the sufficiency for increased intracellular S1P content to induce ASM cell cytostasis.

**FIGURE 7:**
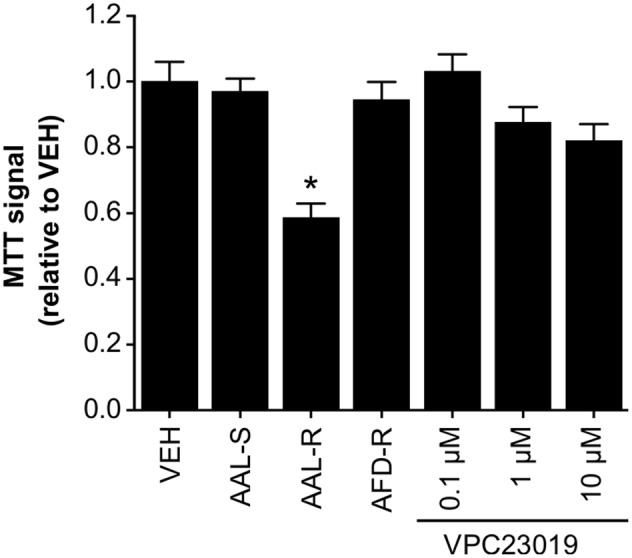
**An S1P_1_/S1P_3_ antagonist does not induce cytostasis.** ASM cells were incubated in complete medium with the AAL-R (1 μM), AAL-S (1 μM), AFD-R (1 μM), VPC23019 (at indicated concentrations) or the vehicle (VEH) for 24 h and cell accumulation was assessed using the tetrazolium dye assay. *n* = 8. ^∗^Shows statistically significant differences from the VEH-treated group at a *p* < 0.05.

Since intracellular accumulation of S1P might reduce extracellular S1P levels and the subsequent impairment of S1P_1_- and S1P_3_-proliferative effects, ASM cells were incubated with the dual S1P_1/3_ antagonist (VPC23019; **Figure 7**). Similar to AFD-R and AAL-S, dual inhibition of S1P_1_ and S1P_3_ failed to significantly affect the accumulation of proliferating ASM cells *in vitro*, when compared to the vehicle.

### AAL-R Incubation Leads to Massive Accumulation of Intracellular AFD-R in ASM Cells

**FIGURE 8:**
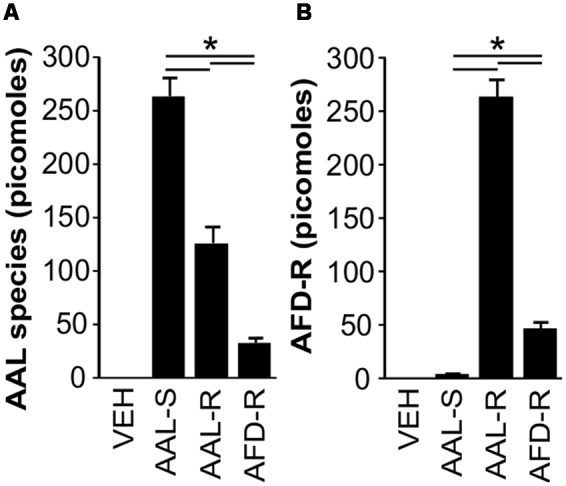
**AAL-R-induced cytostasis depends on intracellular accumulation of AFD-R.** ASM cells were incubated in complete medium. When indicated, AAL-R (1 μM), AAL-S (1 μM), AFD-R (1 μM) or the vehicle (VEH) were added for 24 h. Mass spectrometry was performed to quantify **(A)** unphosphorylated total AAL species and **(B)** AFD-R. *n* = 6. ^∗^Shows statistically significant differences from VEH group at a *p* < 0.05.

Since ASM cells express *SphK2* (Supplementary Figure 2) and because SphK2 activity is required for efficient phosphorylation of AAL-R to AFD-R, we then addressed the hypothesis that AAL-induced ASM cell cytostasis could be explained by intracellular accumulation of AFD-R. Our results show that ASM cells uptake AAL-R and AAL-S preferentially to AFD-R (**Figure 8**). This is demonstrated by the retrieval of relatively high amounts of AAL species in cells incubated with AAL-S (263 ± 17 picomoles) and AAL-R (126 ± 15 picomoles), compared to an equimolar concentration of AFD-R (32 ± 4 picomoles) (**Figure 8A**). Most importantly, in ASM cells, the intracellular balance between the non-phosphorylated and the phosphorylated form of AAL-R favored the accumulation of AFD-R by up to twofold (**Figures 8A,B**). Of note, this AFD-R accumulation (263 ± 16 picomoles) represents more than 100 times the cellular amount of S1P under the same experimental conditions. Thus, ASM cells express *SphK2* and display a massive intracellular accumulation of AFD-R upon incubation with AAL-R.

### AAL-R but not AAL-S Efficiently Reverses AHR

**FIGURE 9:**
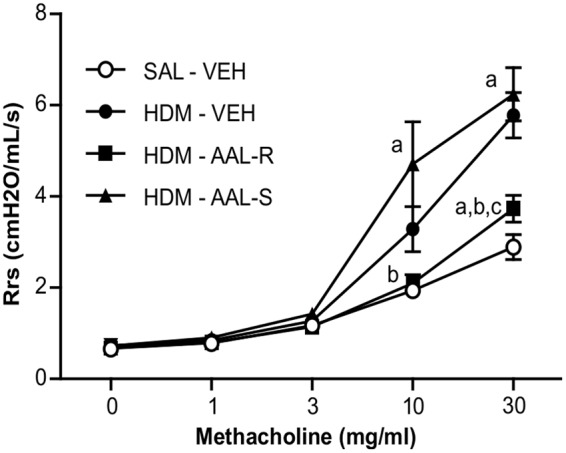
**Reversal of airway hyperresponsiveness induced by chronic HDM exposure requires a phosphorylatable sphingosine analog.** Mice received HDM or saline (SAL) and underwent AAL-R (1 mg/kg) or AAL-S (1 mg/kg) treatment as described in **Figure 2**. The degree of airway responsiveness was assessed by measuring the resistance of the airway system (Rrs) in response to graded doses of methacholine. Saline: *n* = 11; VEH: *n* = 15; AAL-R: *n* = 14; AAL-S: *n* = 6. “a” denotes a significant difference vs. the SAL – VEH group, “b”: vs. HDM – VEH group, “c”: vs. HDM – AAL-S group. Significantly different at *p* < 0.05.

On the basis that efficient cytostasis of ASM cells relies on *in situ* phosphorylation of the sphingosine analog, we ruled out the ability of the non-phosphorylatable sphingosine analog AAL-S to reverse AHR in our *in vivo* model. **Figure 9** depicts normoresponsiveness to graded methacholine doses in mice receiving saline and vehicle. In this group, peak resistance was recorded at 2.89 ± 0.27 cmH_2_O/ml/s in response to an aerosolized dose of 30 mg/ml of methacholine. AHR was present in the group receiving HDM and vehicle, with a threefold increase of respiratory system resistance at 30 mg/ml of methacholine, when compared to mice receiving saline and vehicle. AAL-R again inhibited by more than 70% the HDM-induced AHR at the methacholine dose of 30 mg/ml. In stark contrast, the non-phosphorylatable AAL-S enantiomer failed to alleviate AHR in HDM-exposed mice. Of note, neither AAL-R nor AAL-S affected airway responsiveness to methacholine in mice without experimental asthma (Supplementary Figure 3A), indicating that a 1 week-long exposure to either of these two sphingosine analogs does not influence ASM contractility *per se*. In agreement with the absence of AAL species-induced alterations of lung histology (Supplementary Figure 3B), these results confirm the lack of deleterious effects of these compounds on pulmonary functions over a short period of time.

## Discussion

This is the first evidence supporting sphingosine analogs as proficient molecules to reverse ASM thickening after its instatement, leading to a nearly full functional recovery of AHR in the methacholine challenge test. We also deciphered that immunomodulation is not required for AAL-R to induce a catabolic response in ASM cells, as this effect occurs *in vitro* and in absence of acute inflammation *in vivo*. Still, we also confirmed that the short term AAL-R treatment remained efficient at inhibiting inflammation in the remodeled airways in response to repeated antigen challenges, and failed to induce aversive functional alterations in the lung. We further confirmed that mechanisms of action of this class of agents vary according to their biochemical properties. More specifically, we defined that the efficacy of sphingosine analogs to reverse ASM thickening relied on the propensity to be phosphorylated *in situ* and with the ability to increase intracellular AFD-R levels in ASM cells.

We previously showed that a phosphorylatable sphingosine analog could alleviate hyperreactivity in an acute model of asthma where inflammation, but not remodeling, was pre-established (Gendron et al., 2015). The beneficial effect, similar to what is observed when sphingosine analogs are provided prophylactically, likely resulted from the interference with inflammation-associated release of pro-remodeling/spasmogenic factors (Idzko et al., 2006; Marsolais et al., 2011; Karmouty-Quintana et al., 2012; Collison et al., 2013). Yet in asthma, remodeling is already present and its reversal remains a preclinical challenge as supported by the work of Johnson et al. (2008) showing that three interventions (corticosteroid, beta-2 agonist, and antigen weaning) are required to reverse AHR in a chronic HDM-induced asthma model. These findings are in line with the inability of a high dexamethasone dose (1 mg/kg) to reverse AHR in spite of a significant alleviation of inflammation (Mushaben et al., 2013). In agreement with the loss of efficacy of prophylactic treatments once remodeling is established (Walters et al., 2007; Mushaben et al., 2013), mechanisms that sustain ASM thickening are likely to differ from the ones that initiate its process.

In this regard, ASM cells found in a proliferative environment become resistant to corticosteroid-mediated cytostasis (Stewart et al., 1995; Dixon et al., 1999). In contradistinction, we show a robust anti-proliferative effect of AAL-R occurring at a concentration one order of magnitude lower than its pro-apoptotic/cytotoxic effects. On these bases, it becomes tempting to speculate that the preferential efficacy of AAL-R to inhibit AHR, when compared with dexamethasone, relates to its potency to induce cytostatic/catabolic effects in proliferating ASM cells. This theory also agrees with our observation that the AAL isomer that is not a potent cytostatic agent *in vitro* under mitogenic conditions (AAL-S) fails to reverse AHR *in vivo*, despite its documented anti-inflammatory effects (Collison et al., 2013; Rahman et al., 2016b). Yet, the presumed absence of effect of AAL-S on ASM thickening in our model remains to be confirmed.

According to the pulmonary tropism of this class of compounds (Meno-Tetang et al., 2006), concentrations of AAL-R likely to induce cytostasis are attainable in the lung (Marsolais et al., 2008), supporting a local effect of the sphingosine analog on ASM cells, even when delivered systemically. One limitation of this study is that we were not able to obtain *in vivo* data on the impact of AAL-R on ASM cell proliferation or apoptosis *per se*. While ASM cell proliferation or apoptosis can be successfully assessed in larger mammals (Leung et al., 2004; Ramos-Barbon et al., 2005; Hassan et al., 2010), their assessment remains a challenge in mice due to the small size of the airways, low nuclei count per bronchi, and rapid elimination of apoptotic cells by phagocytes *in vivo* (Thorp, 2010). For these reasons, we can only conclude that the potency of AAL-R to reverse AHR is linked with its propensity to reverse ASM thickening *in vivo* and to induce ASM cytostasis *in vitro*.

Current literature suggests that likely mechanisms underlying the atrophic effects of sphingosine analogs include activation of protein phosphatase 2A (Collison et al., 2013; Rahman et al., 2016a), interference with sphingosine kinase expression/activity (Tonelli et al., 2010), alteration of S1P receptor-driven pro-survival mechanisms (Fuerst et al., 2014) and anti-proliferative/pro-apoptotic effects of intracellular aminophosphate accumulation (Don et al., 2007; Liao et al., 2007; Hagen et al., 2011). Our results support the last possibility. Indeed, our results revoke the involvement of PP2A reactivation in AAL-R-induced cytostasis since potent reduction of ASM cell accumulation *in vitro* was not reproduced by the non-phosphorylatable enantiomer AAL-S, a molecule shown to favor PP2A activation in the context of asthma (Collison et al., 2013). Yet, the possibility exists that AAL-S could inhibit inflammation, when present, in the remodeled airways and by doing so, alleviate AHR. This remains to be investigated. In regards to inhibition of sphingosine kinases, both AAL-R and AAL-S can interfere with sphingosine kinase activity (Don et al., 2007). Yet, only AAL-R efficiently inhibited ASM proliferation *in vitro* (**Figure 5**).

We observed increased intracellular S1P in cells treated with AAL-R. This intracellular S1P likely resulted from interference with S1P lyase activity and/or inhibition of S1P export (Bandhuvula et al., 2005; Hisano et al., 2011). In T cells, inhibition of proliferation was associated with intracellular S1P acting through activation of nuclear S1P_1_ (Liao et al., 2007). Alternatively, inhibition of S1P efflux is also likely to interfere with a positive auto-feedback loop involving S1P receptors (Liang et al., 2013), and might even contribute to counteract pro-remodeling events occurring in response to S1P (Fuerst et al., 2014). Still, the fact that neither the S1P lyase inhibitor SM4, nor the S1P receptor antagonist VPC23019 modulated proliferation of ASM cells argues against these possibilities to explain AAL-R-induced cytostasis. Further studies will be required to examine the intracellular pathways modulated by the different sphingosine analogs. This type of study may provide important insights as to why only AAL-R exerts a cytostatic effect.

It remains possible that differential subcellular localization of accumulating S1P between the current study and previous studies (Liao et al., 2007; Hagen et al., 2009, 2011) might explain the absence of effect of S1P lyase pharmacological inhibition on proliferation. Still, quantification of phosphorylated sphingoid bases in ASM cells show that the intracellular increase of S1P is marginal compared to that of AFD-R in AAL-R-treated cells, with a difference of at least two orders of magnitude in favor of AFD-R. Moreover, the AAL-R-induced increase of intracellular S1P appears to be much lower here than in models where it induces cytotoxic effects (Hagen et al., 2009). Indeed, we found only a threefold increase, while Hagen et al. (2009) showed that S1P lyase genetic knock down caused a more than 100-fold change of intracellular S1P. For these reasons it is more likely that massive accumulation of AFD-R, rather than mild accumulation of S1P, led to ASM cell cytostasis.

The fact that SphK2-metabolized sphingoid bases preferentially interfere with cell accumulation also supports the concept that, as described in Jurkat T cells (Don et al., 2007), intracellular accumulation of AFD-R is the cause of ASM cell cystostasis. For instance, AAL-R is mainly phosphorylated by SphK2 and intracellular AFD-R accumulation is much faster than the accumulation of other sphingosine analogs (like FTY720). Importantly, this faster conversion relates to the preferential ability of AAL-R to interfere with Jurkat T cell accumulation *in vitro*, when compared with FTY720 (Jary et al., 2010). In agreement with these observations, we determined that ASM cells express *SphK2* and display a nearly 2:1 AFD-R to AAL-R ratio. In face of a preferential distribution of SphK2 at the endoplasmic reticulum (Maceyka et al., 2005) and since accumulation of phosphorylated SphK2-derived sphingoid based in that location can interfere with mechanisms that sustain cell survival and promote proliferation through induction of endoplasmic reticulum stress (Hagen et al., 2011; Wang et al., 2015), we conclude that intracellular AFD-R accumulation is the most likely event to explain interference with mechanisms that sustain ASM thickening and the ensuing AHR. Our results do not exclude the possibility that sphingosine analogs not investigated in this study, such as FTY720, could also interfere with ASM cell proliferation and thereby reverse AHR in experimental asthma. However, current literature supports the theory that sphingosine analogs displaying rapid rates of intracellular sphingoid bases accumulation, like AAL-R, are more efficient to interfere with cellular amplification than analogs featuring slower rates of intracellular aminophosphate accumulation (Jary et al., 2010).

It is well-documented that a prolonged daily administration of sphingosine analogs at low doses can increase the risk of pulmonary side effects (Cohen et al., 2010), which likely relates to the immunosuppressive activities of this class of agents. Over a period of time that exceeds months, FTY720 might even be associated with asthma exacerbations as exemplified by two case studies (van Rossum et al., 2014; Zecca et al., 2014). Importantly, such effect was never reported in clinical trials over a short period of time. Nevertheless sphingosine analogs can become highly potent cell death inducers at high concentrations, and potentially harmful if administered at high doses during severe inflammatory conditions (Shea et al., 2010). Still, short term treatments with sphingosine analogs were repeatedly documented not to cause pulmonary damage when administered locally in the airways (Idzko et al., 2006; Marsolais et al., 2009; Gendron et al., 2015). In the current study, we confirm the absence of aversive effects of this type of agents in the airway. In fact, we document that AAL-R remains efficient at inhibiting allergic airway inflammation in the remodeled lung. Given the likely role of proinflammatory mediators in the induction of tissue remodeling (Bates and Maksym, 2011) and because numerous inflammation-derived mediators can favor a spasmogenic response (Bosse, 2012), our findings support the concept that AAL-R might tackle different asthmatic phenotypes by acting on both airway remodeling and inflammation.

## Conclusion

We found that a punctual treatment with AAL-R resorbs the thickening of ASM in the context of asthma, while not aversively affecting the airways within a short time window. Moreover, we determined that AAL-R could directly interfere with ASM cell accumulation *in vitro*. This effect depends on the propensity of this sphingosine analog to be phosphorylated *in situ* and likely results from an increase of the intracellular aminophosphate content. Given the central role of ASM thickening in AHR and asthma, this experimental therapeutic strategy needs to be further explored.

## Author Contributions

Participated in research design: DG, PL, A-ML, M-JB, NF, AD, EB, M-RB, ML, SB, YB, DM. Conducted experiments: DG, PL, A-ML, C-AH, AL-G. Contributed new reagents: SB. Performed data analysis: DG, PL, A-ML, C-AH, AL-G, DM. Wrote or contributed to the writing of the manuscript: DG, PL, A-ML, M-JB, C-AH, AL-G, NF, AD, EB, M-RB, ML, SB, YB, DM.

## Conflict of Interest Statement

The authors declare that the research was conducted in the absence of any commercial or financial relationships that could be construed as a potential conflict of interest.

## Abbreviations

AHR: airway hyperresponsiveness
ANOVA: analysis of variance
ASM: airway smooth muscle
HDM: house dust mite (*D. pteronyssinus*)
PP2A: protein phosphatase 2A
SEM: standard error of the mean
SIP: sphingosine-1-phosphate
S1P_1-5_: S1P receptors 1 to 5
SphK2: sphingosine kinase 2
