# Supplementary material for: A Phosphorylatable Sphingosine Analog Induces Airway Smooth Muscle Cytostasis and Reverses Airway Hyperresponsiveness in Experimental Asthma
Source: Front Pharmacol. 2017 Feb 21;8:78. doi: 10.3389/fphar.2017.00078 (PMC5318459; doi:10.3389/fphar.2017.00078)
Supplement: Supplementary file 1 [file Data_Sheet_1.pdf]

*Supplementary Material*

**A phosphorylatable sphingosine analog induces airway smooth muscle cytostasis and reverses airway hyperresponsiveness in experimental asthma**

***David R. Gendron, Pascale Blais Lecours, Anne-Marie Lemay, Marie-Josée Beaulieu, Carole-Ann Huppé, Audrey Lee-Gosselin, Nicolas Flamand, Anthony S Don, Élyse Bissonnette, Marie-Renée Blanchet, Mathieu Laplante, Sylvain G. Bourgoin, Ynuk Bossé, David Marsolais****

*** Correspondence: David Marsolais: David.Marsolais@criucpq.ulaval.ca**

# 1 Supplementary Material

## 1.1 LC-MS/MS Analysis

Analyses were done on a TripleTOF 5600 (Sciex, Framingham, MA) equipped with an electrospray interface with a 50 µm iD capillary and coupled to an Eksigent µUHPLC (Eksigent, Redwood City, CA). Analyst TF 1.6 software was used to control the instrument, for data processing and acquisition. The source voltage was set to 5.5 kV and maintained at 350°C, curtain gas was set at 25 psi, gas one at 15 psi and gas two at 15 psi. Acquisition was performed in MRM mode. Specific transitions and voltage parameters for each molecule are reported in supplementary Table 1. The gradient was the following 0-0.1min hold 50% B, 0.1-0.5 min from 50% B to 90% B, 0.5-2.5 min from 90% B to 100% B, hold 100% from 2.5-3 min, followed by a 0.5 min post-flush at 35 uL/min at final condition.

Quantification of S1P and sphingosine was done with a standard curve ($R^2$ = 0.998 and 0.989, respectively) and corrected by their respective internal standard. For the quantification of C16 ceramide, AAL-S, AAL-R and AFD-R, the ratio over the C17-Sphingosine as the internal standard was used to calculate absolute amount. To do so, a 5-points standard curve of each molecule was performed by LC-MS/MS to confirm that the ratio of the AUC of the molecules over that of the C17-Sphingosine was linear over the range of observed AUC. Once the confirmation was done, the AUC of the C17-Sphingosine was used to calculate the absolute amount of molecule of interest.

# 2 Supplementary Figures and Tables

## 2.1 Supplementary Table 1: Details of the mass spectrometer parameters

| Molecule | Precursor m/z | MS/MS ion | Declustering potential | Collision energy |
|---|---|---|---|---|
| Sphingosine | 300.3 | 282.28 | 20 | 17 |
| C17-Sphingosine | 286.3 | 268.26 | 20 | 17 |
| S1P | 380.2 | 264.27 | 20 | 23 |
| C17-S1P | 366.4 | 250.27 | 20 | 23 |
| AAL-S/AAL-R | 294.2 | 107.04 | 100 | 25 |
| C16-ceramide | 538.5 | 264.26 | 100 | 39 |
| AFD-R | 375.2 | 260.2 | 100 | 20 |

## 2.2 Supplementary Figures

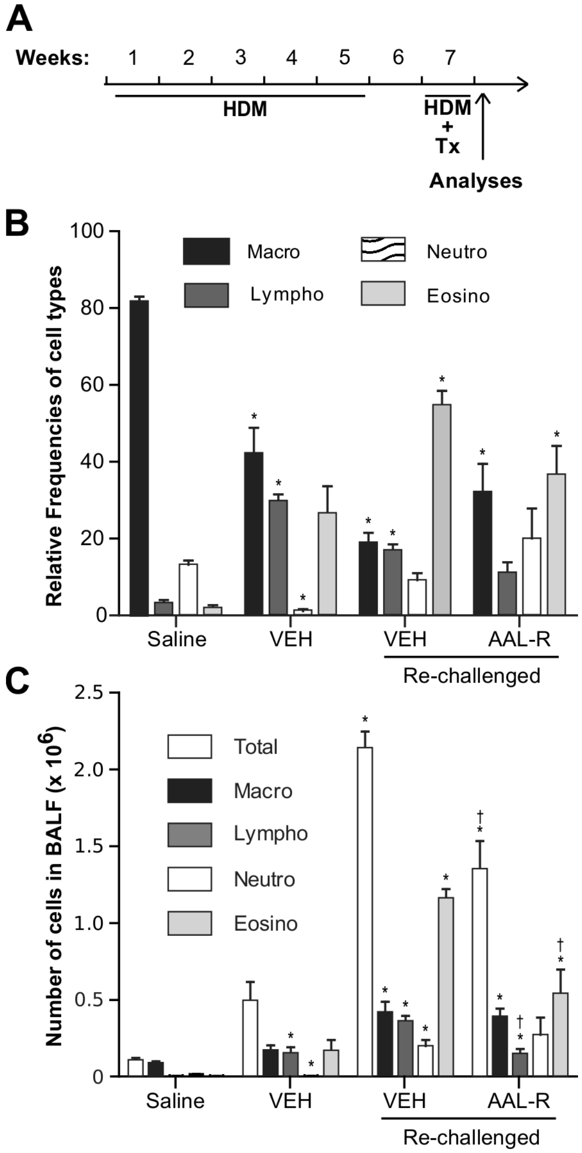


**Supplementary Figure 1: AAL-R retains its ability to alleviate inflammation in the remodelled airways. (A)** Mice received either saline or HDM 3 times a week for 5 consecutive weeks. After 1 week of rest to allow acute inflammation to resolve, mice were re-challenged with HDM i.n or saline. A subset of HDM re-challenged mice were injected i.p. with vehicle (VEH) or AAL-R (1mg/kg) daily for 1 week. Mice were anesthetized 24 h after the last challenge and BALF was harvested for differential counts of Macrophages (Macro), Lymphocytes (Lympho), Neutrophils (Neutro), and Eosinophils (Eosino). **(B)** Relative frequencies of indicated cell subsets. **(C)** Absolute numbers of indicated cell subsets. Mice that were not re-challenged with HDM and received the vehicle were also used as a reference group (Saline). n=6 mice per group. * denotes a significant difference vs the saline group; † denotes a significant difference vs the HDM re-challenged - VEH group, significantly different at $p < 0.05$.

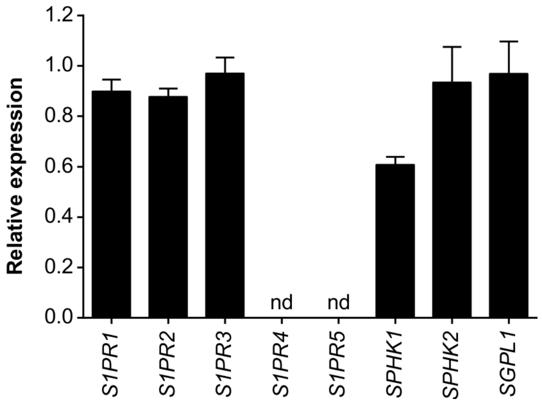


**Supplementary Figure 2: ASM primary human cells express S1P receptors 1 to 3, both sphingosine kinases and S1P lyase.** The expression of **S1P receptors 1 to 5** (*S1PR1*, NM_001400; *S1PR2*, NM_004230; *S1PR3*, NM_005226; *S1PR4*, NM_003775; *S1PR5*, NM_001166215), sphingosine kinase 1 (*SPHK1*, NM_001142601) and 2 (*SPHK2*, NM_020126) and sphingosine-1-phosphate lyase (*SGPL1*, NM_003901) was assessed using real time quantitative PCR. mRNA from human primary ASM cells was extracted using the EZ-10 DNAway mRNA mini-preps kit (Biobasic, Markham, CAN) according to the manufacturer's instructions. mRNA (1µg) was converted to cDNA using iScript Advanced cDNA Synthesis and quantified with the Rotor Gene apparatus using Sso Advanced SYBR Green Supermix (Biorad). Predesigned primer sets (PrimeTime qPCR Assays) were purchased from Integrated DNA Technologies (Coralville, IA). Efficiency was determined for each set of primers and absence of contaminating DNA was confirmed using no-RT controls. The delta-ct was calculated for each target and reported on the geometric mean of the reference Ribosomal protein large p0 (*RPLP0*, NM_ 001002) and Guanine nucleotide-binding protein subunit beta-2-like 1 (*GNB2L1*, NM_ 006098). n=6.

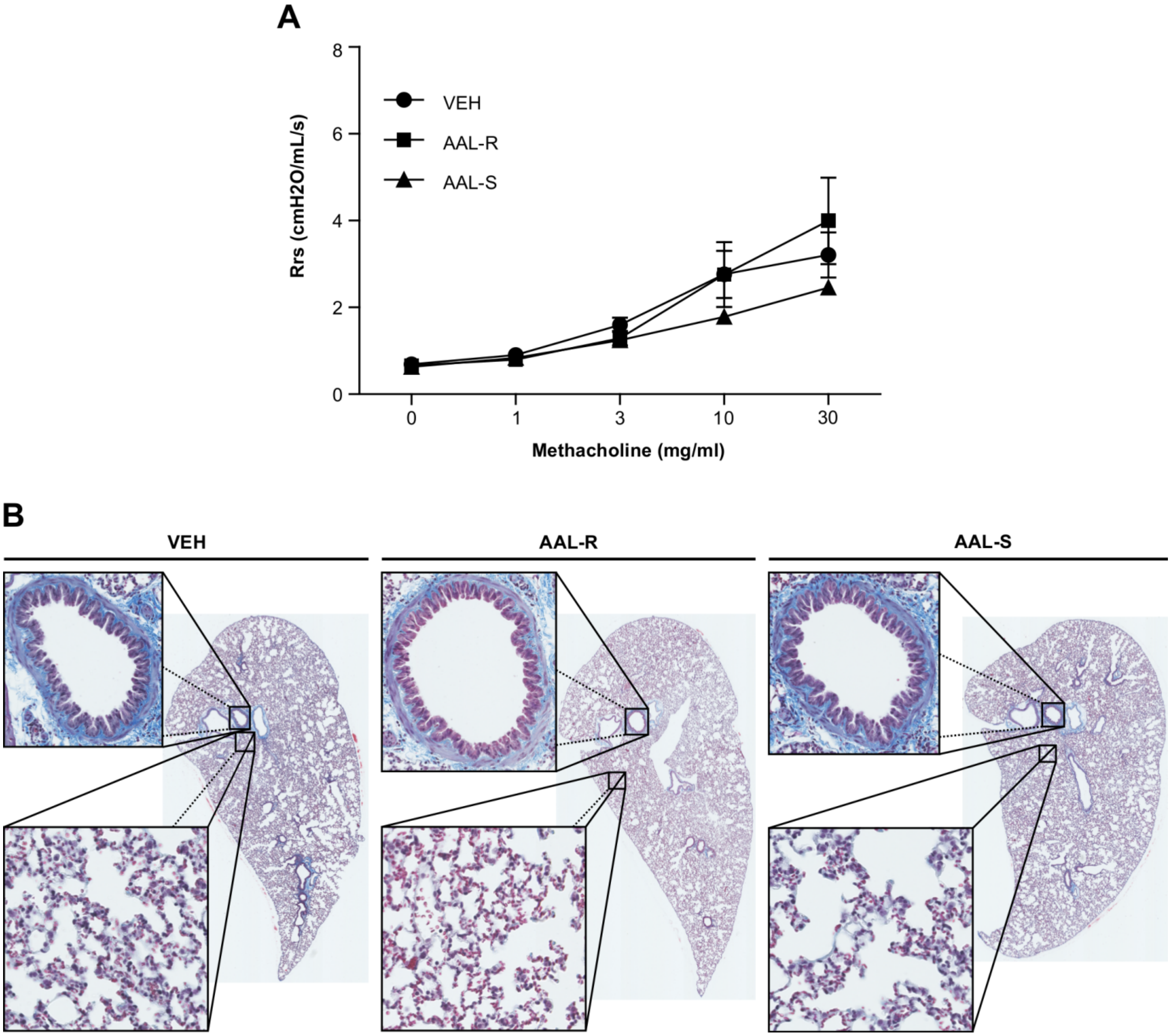


**Supplementary Figure 3: Sphingosine analogs do not negatively affect non-asthmatic lung responsiveness to methacholine.** Naïve mice were administered daily with vehicle (VEH), AAL-R (1mg/kg) or AAL-S (1mg/kg) for 1 week. **(A)** The degree of airway responsiveness was assessed by measuring the resistance of the airway system (Rrs) in response to graded doses of methacholine. **(B)** Histological appearance of sagittal lung slices. n=5. * shows statistical significance from VEH group at a $p < 0.05$.
